# A novel feline herpesvirus vector subunit FCV VP1 and FPV VP2 vaccine protects cats against FHV-1 and FPV challenge and induces serum neutralizing antibody responses against FCV

**DOI:** 10.3389/fimmu.2025.1636514

**Published:** 2025-10-02

**Authors:** Aoxing Tang, Bo Li, Meng Zhu, Shiqiang Zhu, Da Zhang, Na Li, Miao Zhang, Yingqi Zhu, Chuanfeng Li, Chunchun Meng, Jie Zhu, Guangqing Liu

**Affiliations:** Shanghai Veterinary Research Institute, Chinese Academy of Agricultural Sciences, Shanghai, China

**Keywords:** feline parvovirus, feline herpesvirus type 1, feline calicivirus, recombinant virus, vaccine

## Abstract

Vaccines targeting feline parvovirus (FPV), feline calicivirus (FCV), and feline herpesvirus type 1 (FHV-1) are considered core vaccines and are widely recommended for feline immunoprophylaxis. Currently, trivalent feline vaccines used in clinical settings are primarily based on inactivated or modified live attenuated formulations. Among these pathogens, FHV-1 has demonstrated notable potential as a viral vector due to its large genome and immunogenic profile, making it an attractive platform for multivalent vaccine development. In this study, we developed a novel trivalent live attenuated vaccine candidate by engineering FHV-1 as a viral vector to co-express immunogenic proteins from FPV and FCV. Using homologous recombination and CRISPR/Cas9-mediated genome editing, we constructed the recombinant strain FHV ΔgI/gE/TK-FCV VP1-FPV VP2, which expresses FCV VP1 and FPV VP2. Expression of these proteins was confirmed by Western blot and immunofluorescence assay (IFA), and the recombinant virus remained genetically stable during *in vitro* passaging. Immunization with this construct induced robust virus-neutralizing antibody responses and conferred protective immunity against both FHV-1 and FPV. These findings underscore the feasibility of using FHV-1 as a multivalent viral vector and provide a promising foundation for next-generation feline vaccines.

## 1 Introduction

Feline parvovirus (FPV) is one of the most lethal viral pathogens affecting members of the Felidae family, typically presenting with high fever, vomiting, diarrhea, enteritis, and a marked reduction in white blood cell (WBC) count following infection (1). Notably, clinical manifestations vary among infected individuals, with disease severity strongly influenced by factors such as host age, immune status, and concurrent infections. Kittens younger than six months are particularly susceptible, displaying the highest morbidity and mortality rates (2, 3). Taxonomically, FPV belongs to the *Parvoviridae* family and is classified as a non-enveloped, single-stranded DNA (ssDNA) virus with an icosahedral capsid approximately 20–24 nm in diameter. Its genome contains two open reading frames (ORFs), which encode non-structural proteins (NS1 and NS2) and structural capsid proteins (VP1 and VP2) (4). Among these, VP2 is the predominant component, constituting nearly 90% of the viral particle. It plays a dual role by facilitating virion assembly and eliciting host immune responses through the induction of virus-neutralizing antibodies. Importantly, specific amino acid substitutions in VP2 can modulate viral antigenicity and alter host tropism, thereby affecting viral infectivity and immune evasion (5, 6). Given its central role in viral structure and immunogenicity, VP2 has become a key target in the design of recombinant and subunit vaccines against FPV (7, 8).

Feline calicivirus (FCV) and feline herpesvirus type 1 (FHV-1), also referred to as feline viral rhinotracheitis (FVR), are the two most prevalent viral pathogens implicated in upper respiratory tract infections among domestic cats (9–11). FCV, in particular, is highly contagious and demonstrates efficient transmission among felids, leading to a diverse range of clinical presentations—from mild respiratory illness to severe, and occasionally fatal, systemic disease. Notably, infections caused by virulent systemic disease (VSD) strains of FCV (VSD-FCV) can result in mortality rates as high as 50% to 100% (12–14). Genomically, FCV harbors a single-stranded, positive-sense RNA genome comprising three open reading frames (ORFs). ORF1 encodes non-structural proteins essential for viral replication, while ORF2 and ORF3 encode the major and minor capsid proteins, VP1 and VP2, respectively. Among these, VP1 is the principal structural and immunogenic component, recognized by the host immune system and serving as the primary target for the induction of virus-neutralizing antibodies (15, 16). This makes VP1 a key candidate in the development of subunit and recombinant vaccines aimed at controlling FCV infection.

FHV-1, classified as an *α*-herpesvirus, is the principal causative agent of feline viral rhinotracheitis and is responsible for approximately 50% of all viral upper respiratory tract infections in domestic cats (17–19). While cats of all ages can be infected, kittens between two and four months of age are particularly vulnerable, exhibiting near-universal infection rates and mortality rates reaching up to 50%. The clinical manifestations of FHV-1 infection are typically acute and involve both ocular and respiratory systems. Common signs include conjunctivitis, epiphora (excessive tearing), nasal discharge, sneezing, coughing, and anorexia, reflecting the virus’s tropism for the upper respiratory mucosa and conjunctival epithelium (20).

Vaccines targeting FPV, FCV, and FHV-1 are classified as core vaccines and are widely recommended for feline immunoprophylaxis. At present, inactivated vaccines remain the predominant formulations available for clinical use (21). According to the World Small Animal Veterinary Association (WSAVA) guidelines, core vaccinations should be initiated in kittens between six to eight weeks of age, with at least one booster dose administered two to four weeks thereafter (22). Despite their broad use, inactivated vaccines exhibit limited protective efficacy, induce short-lived immunity, and fail to provide complete protection against infections caused by highly virulent respiratory virus strains. Notably, they are ineffective at preventing the establishment of latent FHV-1 infections, a critical gap in disease control (23, 24). Furthermore, conventional vaccines are occasionally associated with adverse effects such as localized swelling, hypersensitivity reactions, anorexia, and fever, which remain unresolved concerns in the field. These limitations underscore the urgent need to develop safer and more efficacious trivalent vaccines capable of simultaneously protecting against FPV, FCV, and FHV-1. In our previous work, we demonstrated that FHV-1 strains harboring deletions in gI/gE or gI/gE/TK genes were non-virulent in kittens and provided robust protection against challenge with virulent FHV-1 (25). Building on this foundation, we further engineered a recombinant FHV-1 strain expressing the functional FCV VP1 protein (FHV ΔgI/gE-FCV VP1), which exhibited an excellent safety profile and conferred strong protection against VSD-FCV, while also inducing a potent immune response (26). In the present study, we employed homologous recombination in combination with the CRISPR-Cas9 genome editing system to construct a novel recombinant FHV-1 strain, designated FHV ΔgI/gE/TK-FCV VP1-FPV VP2. This strain was designed to co-express the major immunogenic proteins FCV VP1 and FPV VP2. We evaluated both the safety profile and immunogenic potential of this recombinant virus in feline hosts. Our findings offer valuable insights into the feasibility of using FHV-1 as a multivalent viral vector, providing a promising platform for future vaccine development against FPV, FCV, and FHV-1.

## 2 Materials and methods

### 2.1 Cells and viruses

Crandell Reese feline kidney (CRFK) cells were cultured in Eagle’s Minimum Essential Medium (EMEM; Wisent, China), supplemented with 10% heat-inactivated fetal bovine serum (FBS; Gibco, USA), 100 µg/mL streptomycin, and 100 IU/mL penicillin. Cells were maintained at 37 °C in a humidified incubator with 5% CO_2_ atmosphere. The FHV WX19 strain is a clinical field isolate obtained from a young cat presenting with severe rhinotracheitis (unpublished) (25). The FPV SH2301 strain was isolated in our laboratory in 2023 from an adult cat that succumbed to feline panleukopenia (unpublished). To generate the recombinant FHV ΔgI/gE/TK eGFP-mCherry strain, CRFK cells were co-transfected with a combination of sgRNA-gI/gE, pT-eGFP, sgRNA-TK, and pT-mCherry plasmids, followed by infection with wild-type FHV-1 as previously described (25). For viral propagation, CRFK cells were infected with the recombinant virus at a multiplicity of infection (MOI) of 0.1. Once cytopathic effects (CPE) were observed in approximately 90% of cells, the culture supernatant was collected and clarified for viral titration. The resulting viral stocks were aliquoted and stored at −80 °C for long-term preservation and downstream applications.

### 2.2 Western blotting

CRFK cells were either mock-infected with EMEM or infected with FHV WX19 or FHV ΔgI/gE/TK FCV VP1-FPV VP2 at a multiplicity of infection (MOI) of 0.5. At 24 hours post-infection (hpi), cells were harvested using lysis buffer (Beyotime, China) for subsequent protein analysis. Protein samples were subjected to SDS-PAGE on 12% gels, followed by transfer to nitrocellulose membranes (GE Healthcare) using a semidry transfer system (Bio-Rad Laboratories). Western blotting was performed to detect the expression of FCV VP1, FPV VP2, gI, gE, TK, and gB proteins, following established protocols (25, 26). β-actin was used as a loading control (25, 27). Membranes were blocked for 2 hours at room temperature in 5% nonfat milk prepared in Tris-buffered saline with 0.05% Tween 20 (TBS-T; 150 mM NaCl, 20 mM Tris, pH 7.3). After blocking, membranes were incubated with the following primary antibodies, all produced in-house unless otherwise noted: rabbit anti-FCV VP1 (1:1000), rabbit anti-FPV VP2 (1:1000), mouse anti-gI (1:1000), mouse anti-gE (1:1000), rabbit anti-TK (1:1000), rabbit anti-gB (1:1000), and mouse anti-β-actin (1:2000; CW Biotech). After five washes (10min each) with TBS-T, membranes were incubated for 1 hour at room temperature with a horseradish peroxidase-conjugated goat anti-mouse IgG secondary antibody (1:10000; Sigma). Protein bands were visualized using enhanced chemiluminescence reagents (Thermo Fisher Scientific) and imaged with an automatic chemiluminescence detection system (Tanon).

### 2.3 Immunofluorescence assay

Monolayers of CRFK cells were seeded in six-well plates and infected with either FHV WX19 or FHV ΔgI/gE/TK FCV VP1-FPV VP2 at a MOI of 0.1. Following a 1-hour incubation at 37 °C, monolayers were washed once with phosphate-buffered saline (PBS), and fresh culture medium was added. Cells were then further incubated for an additional 48 hours at 37 °C prior to IFA analysis to assess the expression of FCV VP1, FPV VP2, and FHV glycoprotein B (gB). At 48 hours post-infection, cells were washed with cold PBS and fixed with 4% paraformaldehyde for 30 minutes at room temperature. After fixation, cells were permeabilized with methanol for 10 minutes at –20 °C, followed by blocking in PBS containing 5% bovine serum albumin (BSA) and 0.3% Triton X-100 for 2 hours at room temperature. Cells were incubated overnight at 4°C with primary antibodies, including anti-FCV VP1 rabbit monoclonal antibody (1:1000, produced in-house) and anti-FPV VP2 mouse monoclonal antibody (1:1000, produced in-house). After washing with PBS, cells were incubated in the dark for 1–2 hours at room temperature with the corresponding secondary antibodies: Alexa Fluor 488-conjugated goat anti-rabbit IgG (H+L) and Alexa Fluor 594-conjugated goat anti-mouse IgG (H+L), both diluted 1:2000 in blocking buffer (5% BSA in PBS). Nuclei were counterstained with 4′,6-diamidino-2-phenylindole (DAPI; Thermo Fisher Scientific) for 4–5 minutes, followed by four additional PBS washes. Fluorescent images were acquired using a Zeiss LSM880 confocal microscope and processed using Zen Blue software (Zeiss, Germany).

### 2.4 Construction of recombinant transfer vectors and single guide RNA plasmids

In previous studies, an expression cassette, pT-FCV VP1, was constructed by replacing the eGFP gene in FHV ΔgI/gE/TK eGFP-mCherry with the FCV VP1 gene via homologous recombination (26). Following a similar strategy, the mCherry gene in the same parental construct was replaced with the FPV VP2 gene. The corresponding expression cassette was designed and assembled in this study. The cassette consisted of the following components: the left homologous arm (Larm1) amplified from the FHV ΔgI/gE/TK eGFP-mCherry genome; the FPV VP2 gene, amplified from cDNA derived from the FPV SH2301 strain (unpublished); the cytomegalovirus (CMV) early promoter sequence, amplified from the peGFP-C3 plasmid (Clontech, USA); and the bovine growth hormone (BGH) polyadenylation signal, amplified from an AAV vector (Clontech, USA). These elements were flanked by the right homologous arm (Rarm1), also amplified from the FHV ΔgI/gE/TK eGFP-mCherry genome. The three purified fragments—Larm1, the FPV VP2 expression cassette, and Rarm1—were seamlessly ligated into the pMD-19T vector (Takara, China) using the ClonExpress^®^ MultiS One Step Cloning Kit (Vazyme Biotech, Nanjing, China). The resulting ligation product was transformed into E. coli DH5α competent cells, and positive clones were screened to obtain the pT-FPV VP2 recombinant plasmid. Plasmid DNA was extracted using standard protocols and subsequently verified by Sanger sequencing. Primer synthesis and sequencing services were provided by TsingKe Biotech Co. Ltd. (Beijing, China).

To facilitate CRISPR/Cas9-mediated gene editing, sgRNAs targeting the eGFP and mCherry open reading frames (ORFs) were designed using the MIT CRISPR design tool (http://crispr.mit.edu). The sgRNA sequences are provided in **Table 1**. The lentiCRISPR v2 plasmid (Addgene) was digested with BsmBI, and specific PCR primers (listed in **Table 1**) were used to amplify the homologous arms flanking the eGFP and mCherry regions from the FHV ΔgI/gE/TK eGFP-mCherry genome.

**Table 1:** Primer sequences used in this study.

Name	Sequences (5’-3’)	Application
gI/gE L arm	F: *AAGCTTGCATGCCTGCAGGTCGAC*GTGGTGGTGTGTGGCATATTA	gI L arm amplification
	R: *GATTACTATTAATAACTA*CTCCAACCTATTTTATGATGG	
gI/gE R arm	F: *ATTGTAAGCGTTAATATTTTG*CTACTTTGAACGTATCGCATC	gE R arm amplification
	R: *TACCCGGGGATCCTCTAGAGA*GCGGCAGCCGCGGTTTATCTA	
TK L arm	F: *AAGCTTGCATGCCTGCAGGTCGAC*GGTTAACGGACGATCTGTGAT	TK L arm amplification
	R: *CCCCGTAATTGATTACTATTAATA*ACTACGTCTGATCTGTGTATGAT	
TK R arm	F: *TAAAGCATTTTTTTCACTG*CACATTAGTGGTGTTCCCT	TK R arm amplification
	R: *TACCCGGGGATCCTCTAGAGA*GCATTCCATCGGCCAGTAATGTATTAG	
FCV VP1 box	F: *CCATCATAAAATAGGTTGGAG*TCCTGCGTTATCCCCTGATTC	VP1 box amplification
	R: *GATGCGATACGTTCAAAGTAG*CAAAATATTAACGCTTACAAT	
FPV VP2 box	F: *CTTTAAAAAACCTCCCACACC*TCCCGTTACATAACTTACGGTAAATGG	VP2 box amplification
	R: *GGTAATAGGGAACACCACTAAT*GTTTAAGATACATTGATGAGTTTGGA	
sgRNA-eGFP	F: CACCGGGCGAGGGCGATGCCACCTA	eGFP sgRNA cloning
	R: AAACTAGGTGGCATCGCCCTCGCCC	
sgRNA-mCherry	F: CACCGGCAACGAGGACTACACCATCG	mCherry sgRNA cloning
	R: AAACCGATGGTGTAGTCCTCGTTGCC	
gI/gE-check	F: CCATCATAAAATAGGTTGGAG	Recombinant virus amplification
	R: GATGCGATACGTTCAAAGTAG	
TK-check	F: GTTTCATCATACACAGATCAGAC	
	R: GGTAATAGGGAACACCACTAATGT	

Bases in italics are the homologous arm for recombination.

### 2.5 Generation of recombinant viruses

To generate the recombinant FHV ΔgI/gE/TK FCV VP1-FPV VP2, CRFK cells were co-transfected with 1 μg each of Cas9 plasmid containing sgRNA-eGFP and sgRNA-TK, along with 2 μg each of the donor plasmids pT–FCV VP1 and pT–FPV VP2, using Lipofectamine 3000 (Thermo Fisher Scientific, USA). Transfection was performed for 18 hours under standard culture conditions. Following transfection, the cells were infected with FHV ΔgI/gE/TK eGFP-mCherry at a MOI of 0.1. Infected cultures were incubated at 37°C in a humidified 5% CO_2_ atmosphere until approximately 90% CPE was observed. Cells were then harvested and subjected to three cycles of freeze–thaw to release viral particles. Recombinant virus was purified from the cell lysates by plaque purification on CRFK monolayers overlaid with 1% low-melting-point agarose and 1% FBS in EMEM. Following seven successive rounds of plaque purification, fluorescent plaques were visualized and enumerated using fluorescence microscopy.

### 2.6 One-step growth curves

CRFK cells were seeded in 96-well plates at a density of 1 × 10^5^ cells per well in 1 mL of EMEM, one day prior to viral infection. The following day, cells were infected with virus at a MOI of 1 and incubated at 37°C for 1 hour to allow viral adsorption. After incubation, the inoculum was removed, and cells were washed three times with 0.01 M phosphate-buffered saline (PBS; pH 7.2) to eliminate unbound virus. Cells were then maintained in EMEM supplemented with 1% fetal bovine serum (FBS) under standard conditions (37°C, 5% CO_2_). At specified time points post-infection, supernatants were collected for subsequent virus titration.

### 2.7 Transmission electron microscopy

CRFK cells infected with either FHV WX19 or FHV ΔgI/gE/TK FCV VP1-FPV VP2 were harvested and fixed in 3% glutaraldehyde for TEM analysis. Following fixation, cell pellets were processed by Wuhan Servicebio Technology Co., Ltd. for ultrathin sectioning and imaging. TEM was subsequently performed to visualize viral particles and assess virion morphology.

### 2.8 Viral challenge in felines

Eight healthy, three-month-old domestic cats, pre-screened and confirmed negative for FHV-1, FPV, and FCV, were randomly assigned to two groups (n = 4 per group). The experimental group received 1 mL of the recombinant FHV ΔgI/gE/TK FCV VP1-FPV VP2 vaccine (10^6^ TCID_50_/mL) via intranasal (IN) immunization, while the control group received 1 mL of cell culture medium via the same route. At 21 days post-immunization (dpi), The experimental group were boosted using the same dose of recombinant virus (10^6^ TCID_50_/mL) via both IN and subcutaneous (SC) routes. On day 42 dpi, both groups were challenged intranasally with FHV WX19 at a dose of 10^5^ TCID_50_/mLL. Clinical signs were monitored daily for 56 days following the primary immunization. Symptom severity was assessed using a previously described scoring system for FHV-1-associated diseases (28), as detailed in **Table 2**. Nasal secretions were collected every three days using sterile polyester swabs. Blood samples were collected weekly, and serum was separated and stored at –20°C for subsequent antibody analysis. All cats were observed throughout the 8-week study period for health status, clinical signs, and immunological response to vaccination and challenge.

**Table 2:** Components of the clinical score.

Category	Signs	Score
Conjunctivitis	Normal	0
	Mild hyperemia	1
	Moderate to severe hyperemia	2
	Moderate to severe hyperemia, with chemosis	3
Blepharospasm	Normal	0
	<25% of eye closed	1
	25–50% of eye closed	2
	50–75% of eye closed	3
	75–100% of eye closed	4
Ocular discharge	75–100% of eye closed	0
	75–100% of eye closed	1
	75–100% of eye closed	2
	75–100% of eye closed	3
Sneezing	Normal	0
	Observed	1
Nasal discharge	Normal	0
	Minor, serous, occasional with blood	1
	Minor to moderate, mucoid or bloody	2
	Marked mucopurulent	3
Nasal congestion	Normal	0
	Mild	1
	Moderate	2
	Severe, with open mouth breathing	3
Coughing	Normal	0
	Coughing noted	1

Eight three-month-old domestic cats, pre-screened and confirmed negative for FHV-1, FPV, and FCV, were randomly divided into two groups (n = 4 per group). One group received 1 mL of the recombinant FHV ΔgI/gE/TK FCV VP1-FPV VP2 virus (10^6^ TCID_50_/mL) via IN immunization, while the control group received 1 mL of sterile cell culture medium by the same route. At 21 dpi, all cats were boosted with 1 mL of the recombinant virus (10^6^ TCID_50_/mLL) via both IN and SC routes. On day 42 dpi, both groups were challenged intranasally with FPV SH2301 at a dose of 10^5^ TCID_50_/mL. Clinical symptoms were monitored daily for 56 dpi. Peripheral blood samples were obtained weekly for antibody analysis, and serum was stored at –20°C until further testing. To assess immune status following FPV challenge, total WBC counts were measured at 42, 49, and 56 dpi using a hemocytometer. Humane euthanasia was carried out in accordance with ethical guidelines for cats exhibiting severe leukopenia, anorexia, and dehydration.

After the end of the experimental procedure, all cats were humanely euthanized in accordance with the “Euthanasia Method for Dogs and Cats” established by the World Animal Protection Society. General anesthesia was first induced via intramuscular injection of Zoletil 50 at a dose of 5 mg/kg. Upon confirmation of complete anesthesia, a 2 mg/kg dose of potassium chloride (KCl) solution was administered intravenously. Death was confirmed by the absence of vital signs and the observation of fixed, dilated pupils. All animal experiments were approved by the Institutional Animal Care and Use Committee of the Shanghai Veterinary Research Institute, Chinese Academy of Agricultural Sciences, and were conducted in full compliance with institutional guidelines.

### 2.9 Real-time PCR on nasal swab extracts

Total DNA was extracted from 200 µL aliquots of each swab sample using the DNeasy Blood & Tissue Kit (QIAGEN, Germany) according to the manufacturer’s instructions. DNA concentrations were determined using a NanoDrop spectrophotometer (Thermo Fisher Scientific, USA). A total of 20 ng of purified DNA was then subjected to quantitative real-time PCR using the 7500 Fast Real-Time PCR System (Applied Biosystems), following previously established protocols (29).

### 2.10 Virus-neutralizing antibody testing

Sera were heat-inactivated at 56°C for 30 minutes and subsequently subjected to serial two-fold dilutions. Equal volumes of diluted sera were mixed with FHV WX19, FPV SH2301, or FCV SH/2014, each at a final concentration of 200 TCID_50_/100 μL. The mixtures were incubated at 37°C for 1 hour to allow virus–antibody interaction. Following incubation, 1 × 10^4^ CRFK cells were added to each well, and the plates were incubated for 72 hours at 37°C in a humidified atmosphere containing 5% CO_2_. CPE were monitored to assess viral neutralization. All samples were tested in duplicate to ensure reproducibility.

### 2.11 Gross pathology and histological, and immunohistochemical examination

Samples of nasal mucosa, tonsil, trachea, lung, retropharyngeal lymph node, heart, brain, liver, kidney and spleen were collected, fixed in 10% buffered formalin, embedded in paraffin, sectioned and routinely stained with HE, and IHC analysis. All sections were examined by a pathologist to evaluate any pathological changes.

### 2.12 Statistical analysis

Before analysis of the data, the normality of repeated measures and the homogeneity of variance were tested with the Shapiro–Wilk test and Levene’s test, respectively using GraphPad Prism Software v6. For the post-attack periods the total clinical scores are shown as the median score for each period and were analyzed using Student’s t-tests and one-way ANOVA tests, again using GraphPad Prism Software v6. For VN antibody titers, the data was log transformed to normalize the data and a two-way ANOVA Tukey’s *post hoc* multiple comparison test was used for analysis. P < 0.05 was deemed to indicate significant differences.

## 3 Results

### 3.1 Construction and identification of the recombinant virus FHV ΔgI/gE/TK FCV VP1-FPV VP2

**Figure 1:**
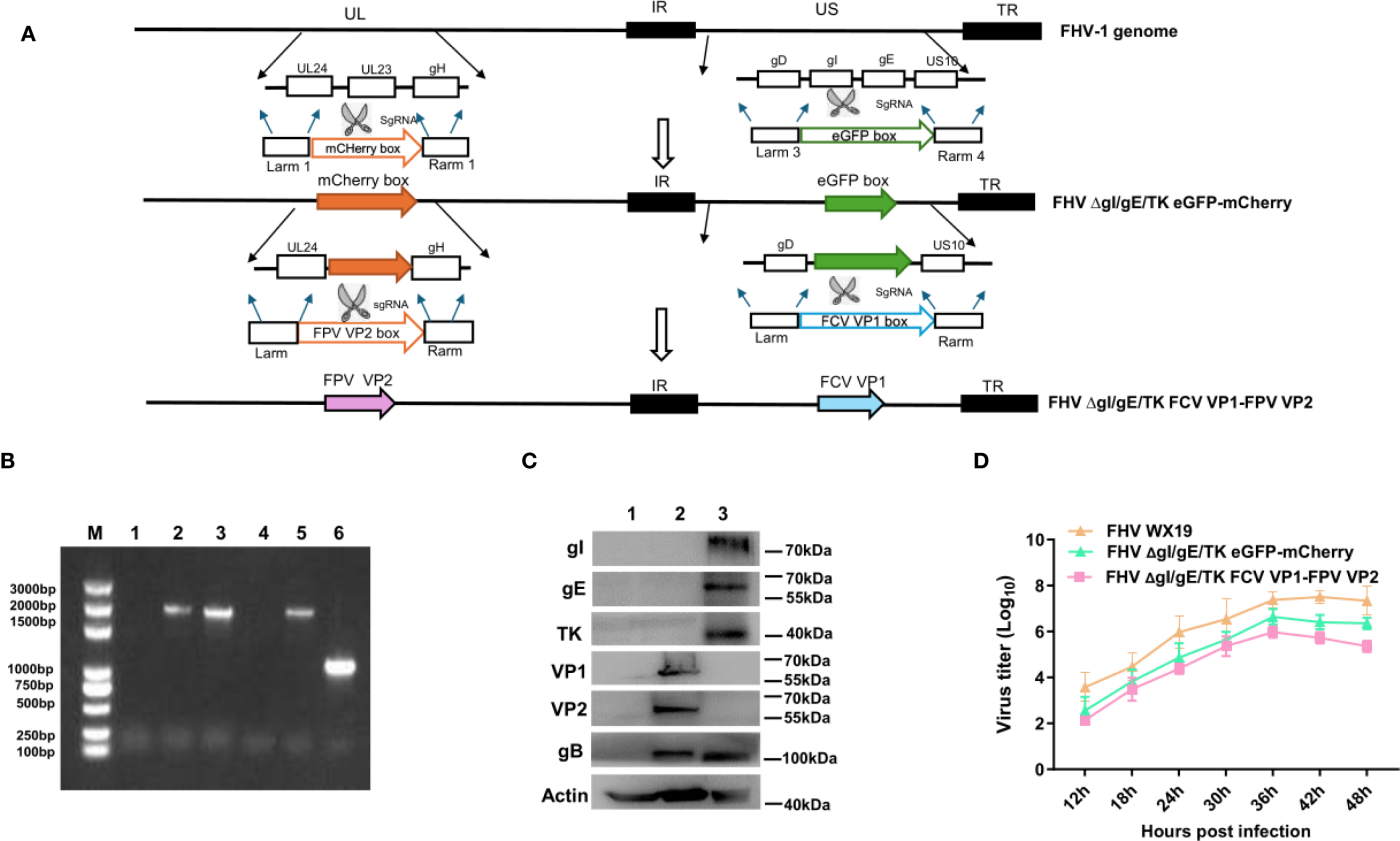
Construction of the recombinant virus FHV ΔgI/gE/TK FCV VP1-FPV VP2. **(A)** The flowchart for construction of recombinant FHV ΔgI/gE/TK FCV VP1-FPV VP2. **(B)** PCR analysis of FHV ΔgI/gE/TK FCV VP1-FPV VP2 using primers gI/gE-check and TK-check, respectively. Lane M, DNA ladder (3,000 bp); Lanes 1–3 are primed with gI/gE-check. Lane 1, CRFK; Lane 2, FHV ΔgI/gE/TK FCV VP1-FPV VP2; Lane 3, FHV WX19. Lanes 4–6 are primed with TK-check. Lane 4, CRFK; Lane 5, FHV ΔgI/gE/TK FCV VP1-FPV VP2; Lane 6, FHV WX19. **(C)** CRFK cells were infected with FHV ΔgI/gE/TK FCV VP1-FPV VP2, FHV WX19 or EMEM as a control and analyzed for the expression levels of VP1 and VP2 proteins. The expression levels of VP1 and VP2 proteins were detected by Western blotting. Lane 1, CRFK; Lane 2, FHV ΔgI/gE/TK FCV VP1-FPV VP2; Lane 3, FHV WX19. **(D)** One-step growth curve of the with FHV WX19, FHV ΔgI/gE/TK eGFP-mCherry and FHV ΔgI/gE/TK FCV VP1-FPV VP2 in CRFK cells.

The FCV VP1 and FPV VP2 expression cassettes were integrated into the FHV ΔgI/gE/TK eGFP-mCherry genome by homologous recombination, using eGFP and mCherry as selection markers as shown in **Figure 1A**, resulting in the creation of the recombinant FHV ΔgI/gE/TK FCV VP1-FPV VP2. The insertion of VP1 and VP2 gene sequences into the FHV ΔgI/gE/TK FCV VP1-FPV VP2 genome was verified by polymerase chain reaction (PCR) using gI/gE-check and TK-check primer pairs, respectively. Recombinant FHV WX19 and FHV ΔgI/gE/TK FCV VP1-FPV VP2 produced different PCR products, as shown in **Figure 1B**. The length of the DNA fragment of FHV ΔgI/gE/TK FCV VP1-FPV VP2 using the gI/gE-check primers was approximately 2600 bp, while the length of the DNA fragment of FHV WX19 was approximately 2700 bp; the length of the DNA fragment of FHV ΔgI/gE/TK FCV VP1-FPV VP2 using the TK-check primers was approximately 2635 bp, while the length of the DNA fragment of FHV WX19 was approximately 1032 bp. The expression of VP1 and VP2 proteins in FHV ΔgI/gE/TK FCV VP1-FPV VP2-infected cells was confirmed by Western blot using polyclonal antibodies against FCV VP1 and FPV VP2, respectively. Bands specifically recognized by VP1 and VP2 polyclonal antibodies were observed in CRFK cells infected with FHV ΔgI/gE/TK FCV VP1-FPV VP2, but not in cells infected with FHV WX19, as shown in **Figure 1C**. The genetic stability of the recombinant virus FHV ΔgI/gE/TK FCV VP1-FPV VP2 was evaluated by *in vitro* passage and plaque purification. By serial passage in CRFK cells for up to 20 generations, the gI/gE-check and TK-check primers for different generations of recombinant viruses consistently amplified only DNA fragments of the target size. PCR fragment sequencing showed that the VP1 and VP2 expression cassettes of the cloned virus were unchanged (results not shown). Using CRFK cells, the *in vitro* growth characteristics of FHV ΔgI/gE/TK FCV VP1-FPV VP2, FHV ΔgI/gE/TK eGFP-mCherry and FHV WX19 were compared. The growth kinetics of FHV ΔgI/gE/TK FCV VP1-FPV VP2 were very similar to those of FHV ΔgI/gE/TK eGFP-mCherry (as shown in **Figure 1D**), indicating that the insertion of FCV VP1 and FPV VP2 expression cassettes had little effect on the *in vitro* proliferation of FHV ΔgI/gE/TK FCV VP1-FPV VP2; and compared with the parental strain FHV WX 19, the activity of FHV ΔgI/gE/TK FCV VP1-FPV VP2 was weakened, which was consistent with the previous results (25).

### 3.2 FHV ΔgI/gE/TK FCV VP1-FPV VP2 Expressed FCV VP1 and FPV VP2 Self-Assembles into VLPs *in vitro*

**Figure 2:**
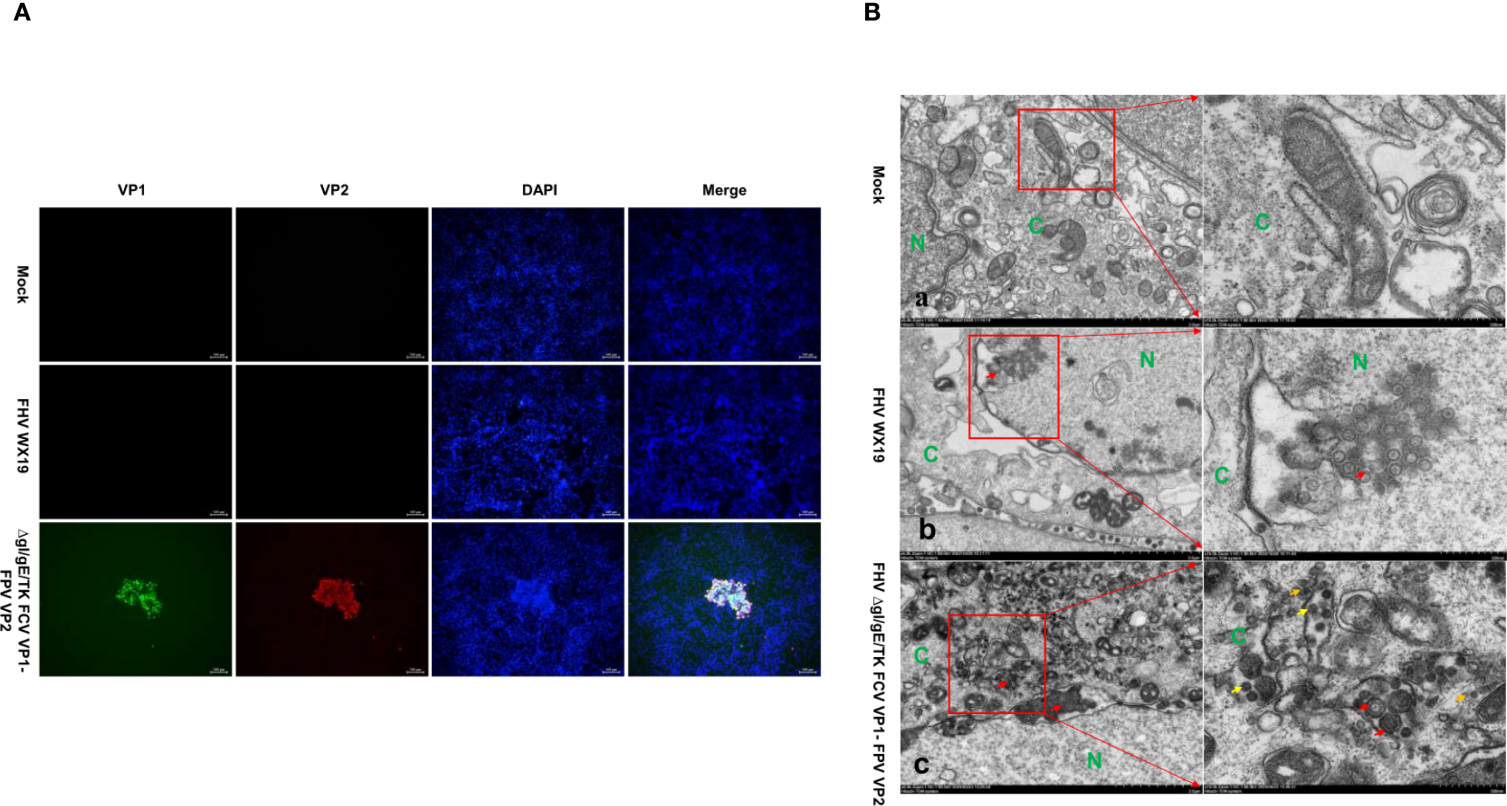
IFA and TEM analysis was conducted on CRFK cells. **(A)** CRFK cells were infected with FHV WX19, FHV ΔgI/gE/TK FCV VP1-FPV VP2 or EMEM as a control and analyzed for the expression levels of VP1 and VP2 proteins. The expression levels of VP1 and VP2 proteins were detected by IFA. **(B-a)** Represents a mock-infected healthy CRFK cell exhibiting normal cellular morphology. **(B-b, c)** CRFK cells infected with FHV WX19 and FHV ΔgI/gE/TK FCV VP1-FPV VP2 (infected at an MOI of 5) were fixed at 18 hours post-infection. **(B-b)** Cells infected with FHV WX19 displayed several fully enveloped FHV virus particles, approximately 100 nm in diameter, within exocytic vesicles in the cytoplasm (red arrows). **(B-c)** Cells infected with FHV ΔgI/gE/TK FCV VP1-FPV VP2 show accumulation of FCV VLPs (yellow arrows) and FPV VLPs (orange arrows) in the cytoplasm. FCV VLPs are round, each with a diameter of approximately 35 nm; FPV VLPs are round, each with a diameter of approximately 24 nm. N, nucleus; C, cytoplasm.

The expression of VP1 and VP2 proteins in cells infected with FHV ΔgI/gE/TK FCV VP1-FPV VP2 was confirmed by IFA. After infection with FHV ΔgI/gE/TK FCV VP1-FPV VP2, CRFK cells showed positive immunofluorescence for VP1 and VP2 proteins, while cells infected with FHV WX19 did not show such fluorescence, as shown in **Figure 2A**. TEM studies were performed to evaluate CRFK cells infected with FHV WX19 and FHV ΔgI/gE/TK FCV VP1-FPV VP2 from a morphological perspective. The primary objective was to identify chimeric FCV and FPV viral particles expressed in cells infected with FHV ΔgI/gE/TK FCV VP1-FPV VP2. Mock-infected healthy CRFK cells exhibited normal cell morphology as shown in **Figure 2B-a**. Cells infected with FHV WX19 displayed characteristic enveloped herpesvirus particles with a diameter of approximately 100 nm within vesicular structures in the cytoplasm as shown in **Figure 2B-b**. When infected with FHV ΔgI/gE/TK FCV VP1-FPV VP2, CRFK cells also showed enveloped FHV virus particles with a diameter of about 100 nm in the cytoplasm at 18 hpi (as shown in **Figure 2B-c**). FCV and FPV VLPs were visible in the cytoplasm. FCV VLPs were round and about 35 nm in diameter, which was consistent with the previous results (26). Each FPV VLP was round and about 24 nm in diameter. Therefore, TEM results confirmed that the VP1 and VP2 capsid proteins expressed by the FHV ΔgI/gE/TK FCV VP1-FPV VP2 vector self-assembled to form VLPs.

### 3.3 FHV ΔgI/gE/TK FCV VP1-FPV VP2 is safe for felins and protects against FHV-1 strains

**Figure 3:**
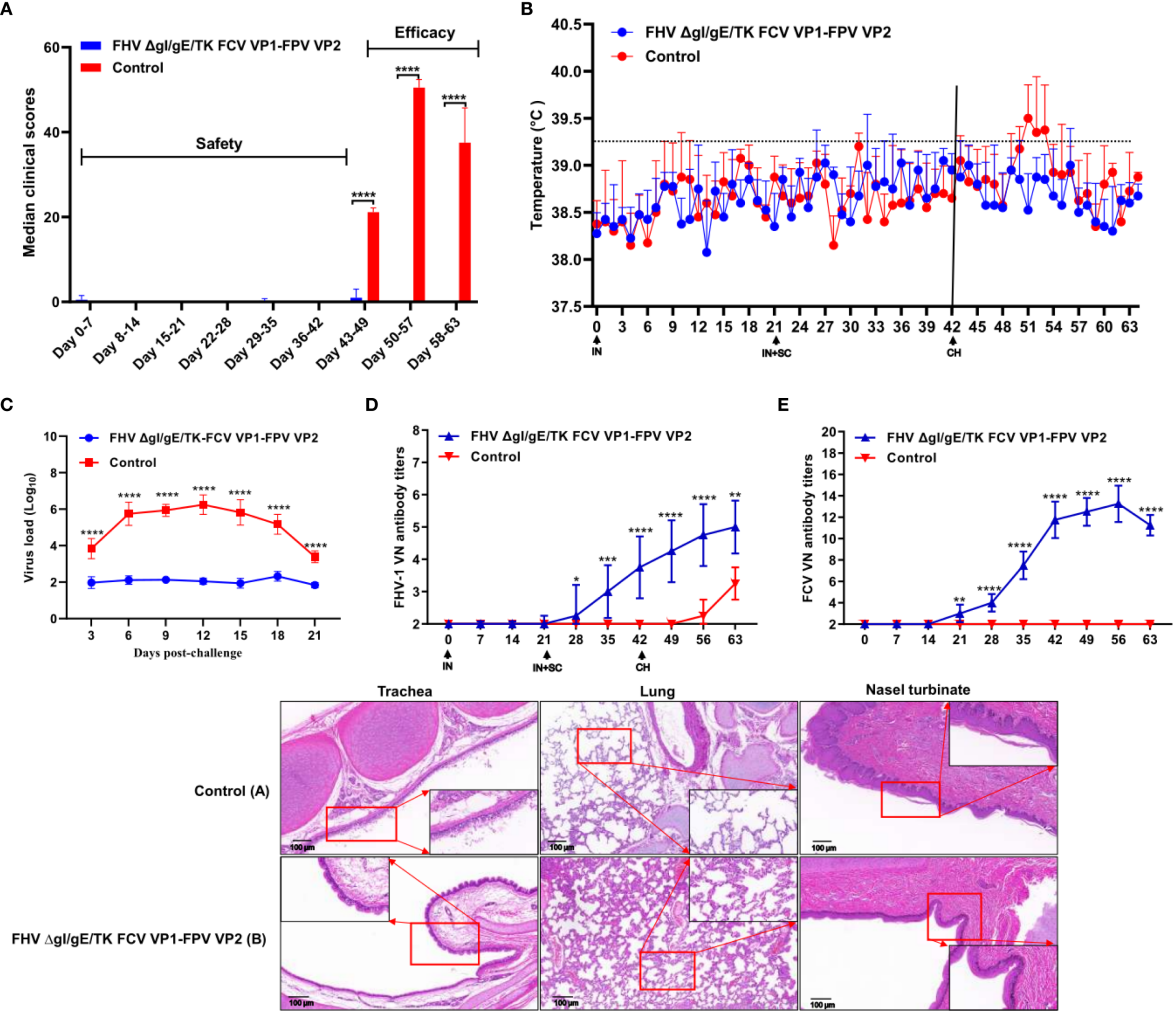
Protection against FHV-1 strains in cats vaccinated with the FHV ΔgI/gE/TK FCV VP1-FPV VP2. **(A)** Median total daily scores of controls are shown after immunization and challenge. Black **** indicate a significant difference (p <0.0001) between the controls and the FHV ΔgI/gE/TK FCV VP1-FPV VP2. clinical scores and standard deviations (SD) for each group (n = 4) are shown. **(B)** Intra-ear temperatures of cats after immunization and challenge. Intra-ear temperatures were measured with a digital thermometer on the indicated days. Mean temperatures and SD for each treatment group are shown. **(C)** Nasal viral shedding in cats following FHV WX19 virus challenge. **(D, E)** Neutralizing Antibody Levels in Cats following Immunization and Challenge. Virus-neutralizing antibodies against FHV-1 or FCV represent the reciprocal of the highest serum dilution inhibiting CPE formation in 50% of wells infected with 100 TCID_50_ of FHV WX19 or FCV SH/2014. **(F)** Histological changes in trachea, lungs, and nasal turbinates at autopsy. Representative images from the control group **(A)**; FHV ΔgI/gE-FCV VP1 group **(B)**. Scale bar is 100 µm. Asterisks denote statistically significant differences between the control group and the groups of three mutants: *P < 0.05; **P < 0.01; ***P < 0.001; ****P < 0.0001.

**Figure 4:**
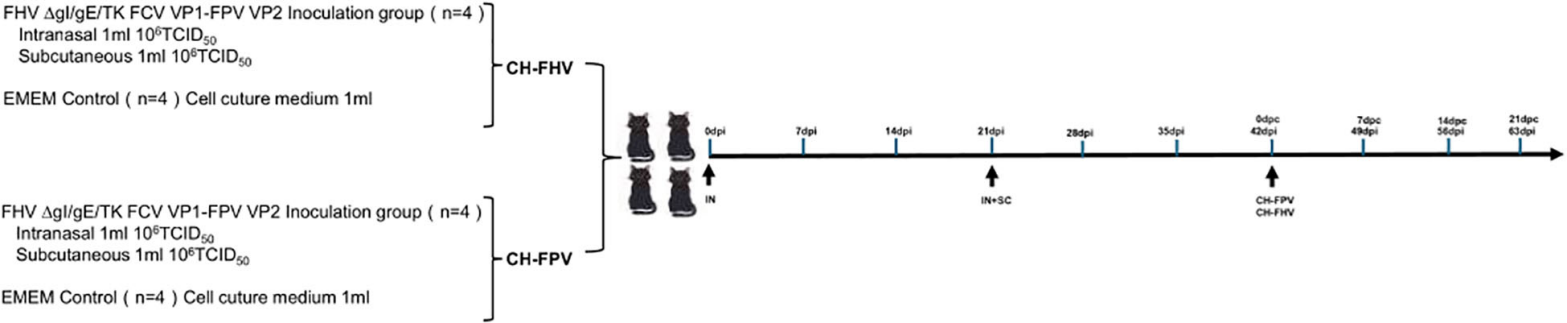
Timeline and experimental design of the study. On days 0 and 21, eight cats in the experimental group were intranasally immunized with FHV ΔgI/gE/TK FCV VP1-FPV VP2. A subcutaneous booster dose was administered on day 21. In contrast, eight control-group cats received no immunization. Subsequently, four cats in the experimental and control groups were vaccinated nasally with virulent FHV WX19 (CH) on day 42, respectively; In addition, four cats in the experimental group and the control group were orally vaccinated with the virulent FPV SH2301(CH) on day 42.

The clinical scores of different experimental groups were assessed following the MSDA scoring system. Prior to immunization, all groups had a clinical score of 0. After the first IN immunization, mild sneezing was observed in one kitten from the FHV ΔgI/gE/TK FCV VP1-FPV VP2 group, lasting for two days. Following the second IN and SC immunization, the symptoms remained similar to those after the first immunization, except for a shorter duration (**Figure 3A**). At 42 dpi, all kittens were challenged IN with the FHV WX19 strain (1 × 10^5^ TCID_50_/mL) (**Figure 4**). During the post-challenge (CH) period, the median total score of the control group exceeded 20. Kittens in the control group exhibited typical clinical signs such as sneezing, excessive tearing, reduced appetite, and conjunctivitis. In contrast, the median total score in the FHV ΔgI/gE/TK FCV VP1-FPV VP2 group was only 4 at 7 days post-challenge (dpc). Overall, the daily clinical scores in the FHV ΔgI/gE/TK FCV VP1-FPV VP2 group were significantly lower than those of the control group (p < 0.0001). Inner ear temperature was measured in all kittens, and no signs of fever were observed before the challenge. At 42 dpi (post-challenge), kittens in the control group exhibited high fever (>39.2°C) (**Figure 3B**), whereas no fever was recorded in any of the vaccinated kittens. Nasal swab samples were collected every three days post-challenge to assess viral shedding using RT-PCR. As shown in **Figure 3C**, the viral load in nasal swabs was significantly lower in the experimental group than in the control group (p < 0.0001). FHV-1 virus-neutralizing antibody (VNA) titers in both experimental and post-challenge groups are presented in **Figure 3D**. One week after the second SC and IN immunization (28 dpi), virus-neutralizing antibodies against FHV-1 began to appear in immunized kittens, with titers gradually increasing until challenge. Similarly, FCV virus-neutralizing antibodies began to emerge in immunized kittens at 21 dpi, reaching a peak titer of 1:215 (**Figure 3E**). Pathological examination was performed at 3 weeks post-infection. The FHV ΔgI/gE/TK-FCV VP1-FPV VP2 group exhibited significantly fewer lesions in the trachea, lungs, and nasal turbinates compared to the control group. In all kittens, lesions were confined to these regions, as shown in the histological images (**Figure 3F**). Control group kittens exhibited mild interstitial pneumonia, characterized by pulmonary congestion, diffuse lung damage, and chronic lymphoplasmacytic rhinitis. In contrast, histopathological changes in the FHV ΔgI/gE/TK-FCV VP1-FPV VP2 group were notably milder, particularly in the nasal turbinates.

### 3.4 Protection against FPV strains in felins vaccinated with the FHV ΔgI/gE/TK FCV VP1-FPV VP2

**Figure 5:**
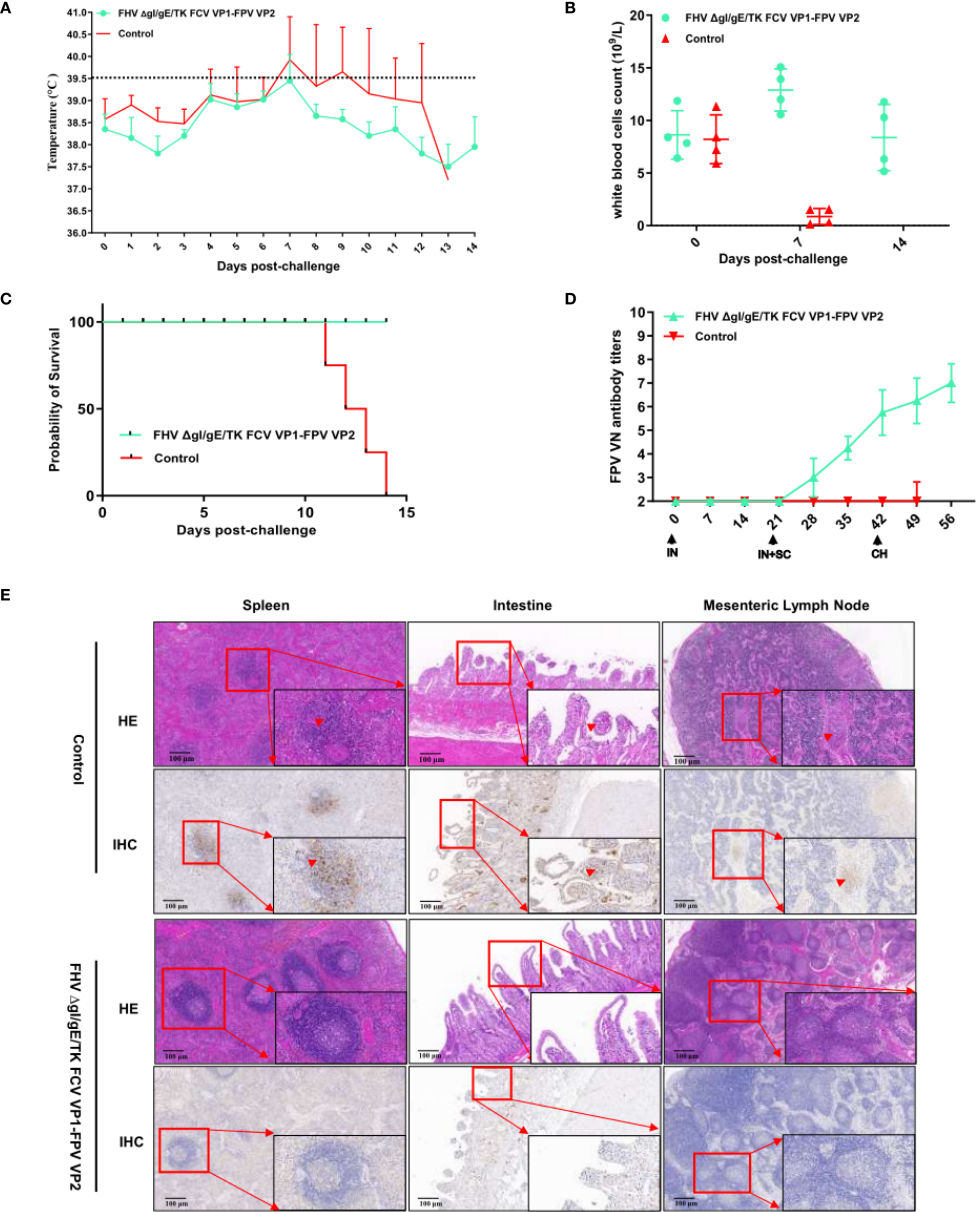
Protection against FPV strains in cats vaccinated with the FHV ΔgI/gE/TK FCV VP1-FPV VP2. **(A)** Intra-ear temperatures of cats after immunization and challenge. Intra-ear temperatures were measured with a digital thermometer on the indicated days. Mean temperatures and SD for the FHV ΔgI/gE/TK FCV VP1-FPV VP2 or EMEM groups are shown. **(B)** During the entire monitoring period after the challenge, blood samples were collected from the FHV ΔgI/gE/TK FCV VP1-FPV VP2 or EMEM groups every 7 days to determine the white blood cells count. **(C)** Percentages of surviving cats after challenge with virulent FPV SH2301. The survival percentages are presented as a Kaplan Meier plot. **(D)** Neutralizing Antibody Levels in Cats following Immunization and Challenge. Virus-neutralizing antibodies against FPV represent the reciprocal of the highest serum dilution inhibiting CPE formation in 50% of wells infected with 100 TCID50 of FPV SH2301. **(E)** HE and IHC changes in spleen, intestines, and mesenteric lymph nodes at autopsy. Scale bar is 100 µm.

All groups of kittens were orally challenged with the FPV SH2301 strain (1×10^5^ TCID_50_/mL) at 42 days post-immunization (dpi) (**Figure 4**). In the control group, all kittens exhibited high fever (>40°C) (**Figure 5A**) and severe clinical symptoms, including depression, vomiting, and hematochezia. In contrast, none of the kittens in the experimental groups developed fever. Venous blood samples were collected from all experimental cats every seven days to assess WBC. As shown in **Figure 5B**, WBC counts remained stable before day 0 post-challenge (dpc); however, by 7 dpc, kittens in the control group experienced a significant decline in WBC counts (WBC < 1.55 × 10^9^/L; normal range: 3.50–19.50×10^9^/L), and all kittens in this group succumbed to infection. In stark contrast, kittens in the FHV ΔgI/gE/TK-FCV VP1-FPV VP2 group survived the viral challenge and remained clinically healthy throughout the observation period (**Figure 5C**). The FPV VNA titers post-challenge are presented in **Figure 5D**. In the FHV ΔgI/gE/TK-FCV VP1-FPV VP2 group, VNA titers began to increase after 28 dpi, with an average pre-challenge titer of 1:64.

Postmortem examination was conducted on both surviving and deceased kittens (**Figure 5E)**. In all deceased kittens from the control group, splenic central arteries and periarteriolar lymphoid sheaths were markedly expanded, accompanied by lymphocyte proliferation. Additionally, necrosis and exfoliation of intestinal mucosal epithelial cells were observed, along with villous atrophy and infiltration of lymphocytes and neutrophils. Mesenteric lymph nodes (MLNs) exhibited hemorrhage and hemolysis. IHC analysis further confirmed FPV infection. Positive immunostaining signals were prominently observed in the spleen, intestine, and small intestinal tissues, characterized by a distinct brown-yellow coloration in the cytoplasm of infected cells, with some nuclei also exhibiting brown-yellow staining.

## 4 Discussion

Currently, trivalent vaccines for feline use in clinical settings are primarily based on inactivated vaccines and modified live attenuated vaccines. Among these, inactivated trivalent vaccines are the most widely administered. However, their protective efficacy is limited, with short-lived immune responses, rendering them insufficient in preventing infection by highly virulent strains responsible for feline upper respiratory tract diseases. Feline herpesvirus type 1 (FHV-1) has shown promising potential as a viral vector platform for vaccine development targeting feline pathogens, offering considerable significance for feline health and disease prevention (30).In recent years, the CRISPR/Cas9 system has been extensively utilized in viral genome editing and vaccine design, enabling precise genetic modifications that enhance vaccine safety and efficacy (31, 32). Within *α*-herpesviruses, gI, gE, and thymidine kinase (TK) are among the most commonly studied virulence-associated genes. Although these genes are not essential for viral replication, their deletion attenuates virulence, making such gene-deleted viral mutants attractive candidates for developing safe and effective vaccine vectors.

In our previous work, we employed CRISPR/Cas9 gene-editing technology to individually delete the gI, gE, and TK virulence-associated genes in the FHV-1 genome. Challenge experiments demonstrated that FHV-1 strains with deletions of gI and gE, or simultaneous deletions of gI, gE, and TK, exhibited excellent safety profiles in cats and provided robust protection against virulent FHV-1 infection (25). To date, most studies on FHV-1-based viral vector vaccines—both domestically and internationally—have focused on the insertion of a single pathogen-specific antigen gene, such as those from FCV, feline immunodeficiency virus (FIV), feline leukemia virus (FeLV), or Rabies virus (33–36). These recombinant FHV-1 constructs have demonstrated varying degrees of immunological protection. Building upon our prior findings, we successfully constructed a recombinant FHV-1 strain expressing a functional FCV VP1 protein (FHV ΔgI/gE-FCV VP1). We evaluated the immunogenicity of the VP1 protein expressed by this strain and confirmed that it elicited a strong immune response, conferred protection against highly virulent FCV infection, and significantly mitigated clinical symptoms in kittens (26).

Currently, research on the insertion of multiple antigen genes into the FHV-1 vector remains limited. Several key factors contribute to this challenge. First, the FHV-1 genome is large and structurally complex, making the simultaneous manipulation of multiple genetic loci technically demanding. Second, the incorporation of multiple antigen genes often requires the replacement of several non-essential regions within the FHV-1 genome. However, inappropriate insertion may adversely affect the virus’s replicative capacity or immunogenic properties. Moreover, the expression levels, immunogenicity, and potential interactions among the inserted antigens can significantly influence the overall immunoprotective efficacy of the recombinant vaccine. Achieving optimal vaccine performance requires careful optimization of gene order, promoter selection, and immunoregulatory strategies to balance the expression of multiple antigens effectively. As a result, the successful integration of multiple highly expressed antigen genes at distinct genomic loci within FHV-1 remains technically challenging, and to date, no comprehensive or systematic breakthroughs in this area have been reported.

In this study, we successfully constructed a recombinant FHV-1 strain, designated FHV ΔgI/gE/TK FCV VP1-FPV VP2, based on a triple-gene deletion mutant lacking gI, gE, and TK. This recombinant virus was engineered to simultaneously express the capsid proteins VP1 of FCV and VP2 of FPV. The expression of both exogenous proteins was validated using WB and indirect IFA, confirming the successful generation of the recombinant virus strain. We further investigated the biological characteristics of the recombinant FHV-1 strain. Growth kinetics analysis revealed that although the replication rate of FHV ΔgI/gE/TK FCV VP1-FPV VP2 was lower than that of its parental strain FHV WX 19, it was comparable to that of the previously constructed FHV ΔgI/gE/TK eGFP-mCherry strain, suggesting maintained replicative competence despite antigenic insertions. Importantly, chimeric capsid proteins FCV VP1 and FPV VP2 expressed in CRFK cells infected with FHV ΔgI/gE/TK FCV VP1-FPV VP2 were shown to self-assemble into virus-like particles (VLPs). These findings indicate that the chimeric capsid proteins retained both structural integrity and antigenic functionality. Moreover, VLPs are known to display densely repetitive surface epitopes, which serve as potent immunostimulatory signals that can be readily recognized by the host immune system (37–39). These properties underscore the immunogenic potential of the recombinant virus and support its application as a multivalent vaccine candidate.

Previous studies have demonstrated that immunization with FHV-1 vaccines via both intranasal (IN) and subcutaneous (SC) routes provides superior protective efficacy compared to SC administration alone. In particular, kittens immunized through a combination of IN and SC routes exhibited greater resistance to clinical manifestations of FHV-1 infection and significantly reduced viral shedding (25, 28). In our current study, we confirmed that the recombinant strain FHV ΔgI/gE/TK FCV VP1-FPV VP2, administered through two IN doses followed by a single SC booster, demonstrated a favorable safety profile. Only one out of four kittens in the experimental group exhibited mild clinical symptoms post-immunization, with no fever observed in any animals. Compared with the unvaccinated control group, kittens vaccinated using the FHV ΔgI/gE/TK FCV VP1-FPV VP2 regimen showed markedly improved clinical protection against FHV-1 challenge. These findings align with established knowledge that FHV-1 vaccines confer protection primarily through the induction of both virus-neutralizing antibodies and cell-mediated immunity, and that serum antibody levels alone are not always predictive of protection (40, 41). As expected, kittens immunized with FHV ΔgI/gE/TK FCV VP1-FPV VP2 began to produce FHV-1 neutralizing antibodies by 21 dpi, with titers gradually increasing prior to viral challenge. In parallel, the production of FCV-neutralizing antibodies was also detected in the experimental group after 21 dpi, indicating successful antigen expression and immune activation. Importantly, the results also confirmed that the insertion of exogenous genes (FCV VP1 and FPV VP2) did not interfere with the replication competency or immunogenicity of the herpesvirus vector in the host. This observation is consistent with previous findings (26), further supporting the feasibility of using FHV-1 as a versatile viral vector for multivalent vaccine development in felines.

At present, numerous studies have explored the development of vector-based vaccines against FPV; however, the majority remain at the experimental or preclinical stage. For example, the use of recombinant raccoonpox virus (RCNV) to express the FPV VP2 open reading frame has been shown to confer protective immunity against FPV challenge (42, 43). Similarly, recombinant rabies virus and canine adenovirus-based vaccines expressing FPV VP2 have demonstrated complete protection against virulent FPV infection (1, 44). In the present study, all cats in the unvaccinated control group exhibited severe clinical signs following FPV challenge and ultimately succumbed to infection. In stark contrast, cats in the FHV ΔgI/gE/TK FCV VP1-FPV VP2 group survived the challenge and remained clinically healthy throughout the entire observation period, indicating strong protective efficacy. In our study, prior to challenge, the mean neutralizing antibody titer against FPV reached 1:64. Although this titer was not exceedingly high, it exceeded the threshold required for protective immunity. Taken together, these findings clearly demonstrate that the FHV ΔgI/gE/TK FCV VP1-FPV VP2 recombinant strain is both safe and effective in cats, a conclusion further supported by the survival and health outcomes observed following FPV challenge.

Currently, trivalent feline vaccines used in clinical practice are primarily based on either inactivated or modified live attenuated (MLA) virus platforms. Although these vaccines offer a degree of protection, they exhibit well-documented limitations. Inactivated vaccines generally elicit weak cellular immune responses and are often insufficient to prevent viral infection or shedding, particularly for FHV-1 and FCV. On the other hand, while MLA vaccines are more immunogenic, they pose a risk of reversion to virulence and have been linked to adverse effects, including localized swelling, fever, and hypersensitivity reactions. Furthermore, current formulations fail to completely block latent FHV-1 infection or prevent clinical disease caused by virulent FCV strains.

In contrast, the recombinant trivalent vaccine developed in this study—FHV ΔgI/gE/TK-FCV VP1-FPV VP2—presents several key advantages. It utilizes an attenuated FHV-1 vector backbone engineered for safety through CRISPR/Cas9-mediated deletion of the gI, gE, and TK genes, thereby minimizing the risk of reversion and reducing pathogenicity. This platform enables simultaneous expression of the FCV VP1 and FPV VP2 capsid proteins, both of which self-assemble into VLPs—a structural feature absent in current commercial vaccines. These VLPs display highly repetitive antigenic surfaces that are known to enhance B-cell receptor crosslinking and promote strong humoral immune responses. Additionally, the combined IN and SC immunization routes are designed to elicit both mucosal and systemic immunity, thereby improving protective coverage at primary sites of viral entry—a recognized limitation of traditional injectable-only vaccines.

In summary, the FHV-based recombinant trivalent platform represents a promising next-generation alternative to existing feline vaccines. Its improved safety profile, VLP-mediated immunogenicity, and dual-route delivery system collectively support its potential for broader and more effective protection. Future studies involving head-to-head comparisons with commercial products, expanded challenge trials, and field evaluations will be essential to confirm its clinical applicability and translational value.

## 5 Conclusion

In conclusion, we successfully engineered a novel recombinant FHV-1 strain (FHV ΔgI/gE/TK FCV VP1-FPV VP2) capable of co-expressing the VP1 protein of FCV and the VP2 protein of FPV. Our findings further revealed that the insertion of these exogenous antigen genes did not compromise the virulence attenuation or immunogenicity of the FHV-1 recombinant vector. More importantly, immunization with FHV ΔgI/gE/TK FCV VP1-FPV VP2 induced robust neutralizing antibody responses and effectively activated protective immunity against both FHV-1 and FPV. These results position this recombinant virus as a promising trivalent vaccine candidate for the prevention and control of FPV, FCV, and FHV-1 infections in felines. Thus, the outcomes of this study provide valuable experimental evidence to support the future development of multivalent vaccines targeting major feline viral pathogens.

## Data Availability

The datasets presented in this article are not readily available because or privacy concerns. Requests to access the datasets should be directed to liugq@shvri.ac.cn.
